# Chemical Synthesis of Bacteriophage G4

**DOI:** 10.1371/journal.pone.0027062

**Published:** 2011-11-16

**Authors:** Ruilin Yang, Yonghua Han, Yiwang Ye, Yuchen Liu, Zhimao Jiang, Yaoting Gui, Zhiming Cai

**Affiliations:** 1 Guangdong and Shenzhen Key Laboratory of Male Reproductive Medicine and Genetics, Institute of Urology, Peking University Shenzhen Hospital, Shenzhen PKU-HKUST Medical Center, Shenzhen, China; 2 Guangzhou Panyu Central Hospital, Guangzhou, China; 3 Shantou University Medical College, Shantou, China; 4 Urogenital Institute of Shenzhen University, Shenzhen Second People's Hospital, Shenzhen, China; 5 Shenzhen Second People's Hospital, Shenzhen, China; Charité-Universitätsmedizin Berlin, Germany

## Abstract

**Background:**

Due to recent leaps forward in DNA synthesis and sequencing technology, DNA manipulation has been extended to the level of whole-genome synthesis. Bacteriophages occupy a special niche in the micro-organic ecosystem and have potential as a tool for therapeutic agent. The purpose of this study was to carry out chemical synthesis of the bacteriophage G4 and the study of its infectivity.

**Methodology/Principal Findings:**

Full-sized genomes of bacteriophage G4 molecules were completed from short overlapping synthetic oligonucleotides by direct assembly polymerase chain reaction and ligase chain reaction followed by fusion polymerase chain reaction with flanking primers. Three novel restriction endonuclease sites were introduced to distinguish the synthetic G4 from the wild type. G4 particles were recovered after electroporation into Escherichia coli and were efficient enough to infect another strain. The phage was validated by electron microscope. Specific polymerase chain reaction assay and restriction analyses of the plaques verified the accuracy of the chemical synthetic genomes.

**Conclusions:**

Our results showed that the bacteriophage G4 obtained is synthetic rather than a wild type. Our study demonstrated that a phage can be synthesized and manipulated genetically according to the sequences, and can be efficient enough to infect the Escherichia coli, showing the potential use of synthetic biology in medical application.

## Introduction

Bacteriophages, being viruses that infect bacteria, have played an important role in underpinning the development and advancement of the biosciences since the dawn of molecular biology. Bacteriophages, first discovered around 1915, occupy a special place in viral biology. They are perhaps the best understood viruses. Their genome being less than 10,000 bases long is particularly amenable to genetic alterations at the level of whole genome synthesis thanks to the recent advance made in DNA synthesis and sequencing technology. We may now modify genetic information to an extent not possible before. Due to the advances in synthetic biology, it is now possible to obtain large segments of synthetic DNA, assemble them into entire genomes of infectious agents, and boot them to life. Cello J et al [1] achieved the first chemical synthesis of a DNA (7,500 bp) corresponding to the entire genome of poliovirus. The DNA of the poliovirus was the largest DNA sequence ever synthesized in 2002. By establishing conditions for assembly of the genome, Venter improved upon the methodology and completed the infectious genome of bacteriophage ΦX174 (5,386 bp) within 2 weeks [2]. Researchers further made a distinct improvement in the scale of synthesis which was the 582,970 bp genome of Mycoplasma genitalium [3]. Once again, Venter and his colleagues succeeded in creating the first living organism with a completely synthetic genome [4]. A few medical applications of chemical genome synthesis also have been described [5], [6]. The common goal of this new strategy is to further our understanding of an organism's properties and to make use of this new information to prevent or treat human disease.

Bacteriophage G4, first isolated in 1973 [7], is a genus of Escherichia coli (E. coli) phages of the family Microviridae. G4 or ΦX174-like, is an icosahedra particle with almost the same density as bacteriophage ΦX174 which has been used in many landmark experiments, and contains single-stranded circular DNA [8]. The 5,577 nucleotide long sequence of bacteriophage G4 DNA has been determined, with an average of 66.9% nucleotide sequence similarity compared to that of bacteriophage ΦX174 [9]. These, along with the successful whole genome assembly of bacteriophage ΦX174, can validate G4 as an attractive target for chemical synthesis in medical use.

G4 can only multiply within E. coli and kill the cells by lysis. E. coli are the most common etiologic agent clinically associated with Urinary Tract Infection (UTI) which is the most frequently diagnosed kidney and urologic disease [10], [11]. However, there is documentation of increases multidrug-resistant pathogens [12]. For antibiotic-resistant infection, phage therapy may have potential uses in human medicine [13]. Chemical synthesis of G4 genomes will provide a new perspective of medical application.

All these facts induced us to carry out the synthesis of the ΦX -like phage and the study of its infectivity. We set out to generate a synthetic G4 genome (syn-G4) by whole genome assembly. To tell the difference from the wild type of the phage G4, we genetically alter 3 restriction endonuclease sites during the synthesis of the genome and subsequently generate a mutated G4 genome (m-G4). Two of these infectious circlular genomes produced were electropotated into DH5α and the plaques of syn-G4 and m-G4 were observed after incubation. Both of the phages were confirmed and were able to infect E. coli strain BL21. Our report demonstrates that an infectious phage can be synthesized and manipulated genetically according to the sequence published, and efficient enough to infect the E. coli, showing the potential use of synthetic biology for medical application.

## Results

### Chemical synthesis of syn-G4 and m-G4 genomes

**Figure 1:**
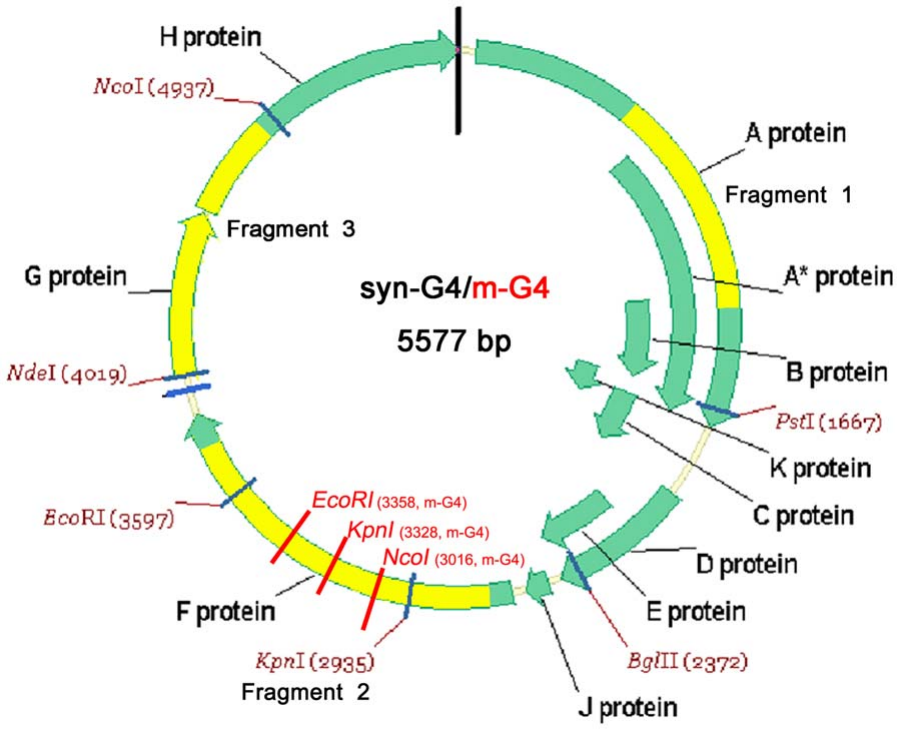
Structure drawing of single-stranded circular syn-G4/m-G4 genome (5577 bp) and sites of restriction endonucleases. New NcoI, KpnI and EcoRI sites marked in red slash were added into the m-G4 genome. Three specific PCR fragments were shown in yellow.

The oligonucleotides were designed to synthesize a G4 genome with exactly the same sequence reported by Godson et al. in 1978 [9] [NCBI Reference Sequence: NC_001420.2]. The sequence was designed to make a molecule that could be cleaved to size with *PstI* and then circularized to produce a circular molecule (Fig. 1).Three restriction endonuclease sites (*NcoI*, *KpnI* and *EcoRI*, respectively) were introduced to generate a m-G4 genome. Since the mutation sites are located at the third base of the corresponding codon, minimal structural and functional impact was expected. The oligonculeotides were pooled and gel-purified by Sangon Biotech as described in Materials and methods. The sequences of the short custom-made segments were consistent with the results from both sequencing and agarose gel. Full-sized genomes of syn-G4 and m-G4 molecules were completed and provided by Shanghai Sangon Biotech. Co., Ltd (data shown in Material S1).

### Circularization of full-length genome and infectivity testing

**Figure 2:**
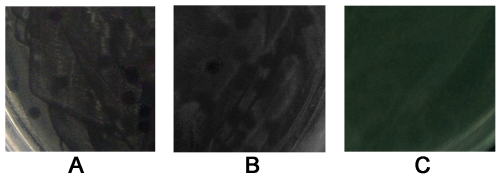
Close up images of the plaques (A, syn-G4; B, m-G4) and the bacterial lawns without plaques (C, control). The plaques appeared small, clear and round. Some merged or ran together to form a mass. The appearance of the plaques of both syn-G4 and m-G4 showed no difference in A and B (A, syn-G4; B, m-G4).

The polymerase chain reaction (PCR) productions of full-length genomes were cleaved with *PstI* and then circularized by ligation with T4 ligase using recommended conditions to generate products of syn-G4 and m-G4 circular molecules, then each 10 µl of ligation product was electroporated into DH5α cells, immediately diluted with 600 µl of liquid Luria-Bertani (LB) medium, and then divided into two screwcapped glass culture tubes. The tubes were rotated at 37°C for 60 min and then 200 µl of culture fluid was stained to LB solid culture medium to stay overnight at 37°C. Phage plaques were visualized after 6–12 h of incubation at 37°C (Fig. 2, A, syn-G4; B, m-G4). The phage formed small, clear, round plaques and some merged or ran together to form a mass on the LB lawn. 10 µl of pure water underwent the same procedures for control group. No plaque was observed after incubation at 37°C (Fig. 2, C, control). Phage from the plate underwent plaque purification and 100 µl of phage suspension with an estimated concentration of 10^−5^/L was added to 100 µl of log phase BL21. Phage plaques were visualized incubation of 6–10 h but formed smaller plaques compared to which generated by with a same concentration.

### Morphology by electron microscope

**Figure 3:**
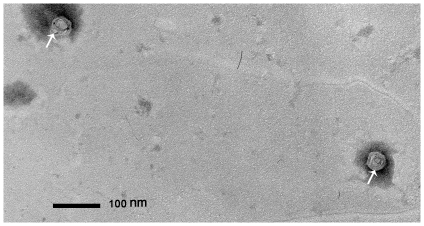
Synthetic G4 morphology by transmission electron microscope (Arrow). Magnification: 800,000×. Bar: 100 nm.

Synthetic G4 samples were positive stained with 0.2% (w/v) phosphotungstic acid and examined by transmission electron microscopy, as seen in Fig. 3. The phage looked like an icosahedral protein shell and the shape was seen approximately 50 nm in diameter (Average of anteroposterior diameter and left-right diameter).

### PCR assay and restriction analyses

**Figure 4:**
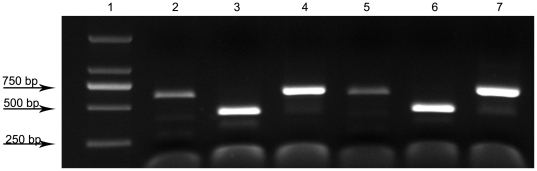
PCR of specific sequences. Agarose gel showing the PCR results obtained when specific primers were used. Lane 1, D2000 marker; Lane 2, 3 and 4, PCR amplification of the three specific products 603 bp, 443 bp and 645 bp, respectively, of syn-G4 (Location shown in Fragment 3, 1 and 2 in yellow in Fig. 1, respectively); Lane 4, 5 and 6, PCR amplification of the three specific products 603 bp, 443 bp and 645 bp, respectively, of m-G4 (Location shown in Fragment 3, 1 and 2 in yellow in Fig. 1, respectively).

**Figure 5:**
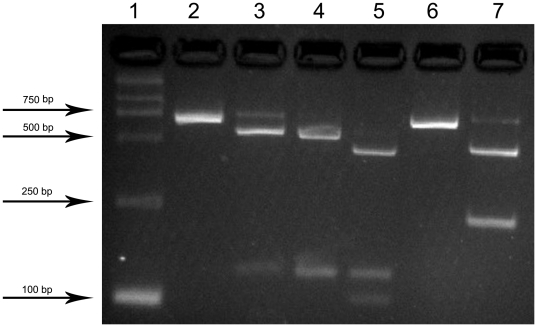
Digestion of restriction endonuclease. Lane 1, D2000marker. Agarose gels (Lane 2, 4, 6) showing the products (shown in Fragment 2 in Fig. 1) of syn-G4 digested by restriction endonucleases and generations of restriction endonuclease digestion fragments of 645 bp (*EcoRI*), 125 bp+520 bp (*KpnI*) and 645 bp (*NcoI*), respectively. The 3 modified restriction endonuclease sites of m-G4 were confirmed by agarose gels and different digestion fragments of 518 bp+127 bp (*EcoRI*), 125 bp+423 bp+97 bp (*KpnI*), and 209 bp+436 bp (*NcoI*) (Lane 3, 5 and 7 respectively), were observed.

Three different parts of the genome were targeted for PCR assay, considering each part coding important structural proteins. The three specific PCR fragments, shown in yellow in Figure 1, of both of syn-G4 and m-G4 were successfully amplified from plaques purification using the specific primer pair (Tab. 1) and confirmed with the results of sequencing. The resulting PCR product was subjected to agarose gel electrophoresis (Fig. 4). The 645 bp PCR amplification products of both syn-G4 and m-G4 were validated by sequencing to ensure the sequences without mutations and including the previous 3 restriction endonuclease sites (data shown in File S1). The 645 bp sequence of syn-G4 contained only 1 *KpnI* site. These sequences of m-G4 contained 2 *KpnI* sites, 1 *NcoI* site and 1 *EcoRI* site after genetic alteration shown in red in Figure 1 with slash at the position of 3016, 3328 and 3358 respectively. The 645 bp DNA products were subjected to restriction enzyme digestion with *EcoRI*, *KpnI* and *NcoI* to excise different segments according to the supplier's recommendations. Subsequently, m-G4 could be digested by *EcoRI*, *KpnI* and *NcoI* respectively, but syn-G4 just could be digested by *KpnI* (Fig. 5). Consistent with the sequencing results, the genetic alteration sites were confirmed by restriction enzyme digestion.

In this report, the natural DNA was unavailable to the experimenter. A chemical synthesis of a circular G4 genome was transfected into E. coli, which produced viable bacteriophages and caused E. coli lysis. To distinguish from the wild type G4, m-G4 was genetically modified with 3 restriction endonuclease sites, which subsequently turned out to be an infectious phage after transfection into E. coli. Both of the plaques could not be identified in morphology, but there were obvious differences observed after digestion of genomes with 3 restriction endonucleases. These results demonstrate that the phages causing lysis of E. coli. were originally from our designed syn-G4 and m-G4 ones. The difference between G4 and M-G4 showed that this isolate is synthetic rather than a wild type. The chemical synthesis of genome was efficient enough to form infectious molecules and subsequently infect the E. coli.

## Discussion

The result in this report proves the correctness of Godson's sequence meaning that is accurate enough to produce an infectious phage. The genetic modification of syn-G4 subsequently generating m-G4 validates the capability of manual manipulation. The development of synthetic approach is still at an early stage, but this field of research has attracted much attention [14], [15]. The methodology of synthetic biology has been improved, from forming an infectious agent to a cellular genome with high efficiency and yield. Apart from the resurrection of the 1918 influenza virus and the generation of codon- and codon pair-deoptimized polioviruses, researchers have reengineered bacteriophages to combat antibiotic-resistant bacteria by endowing them with genetic mechanisms that destroy bacterial mechanisms for evading antibiotic action [16], [17]. Therefore, phage therapy, especially using synthetic phages, might be an alternative method to control and combat pathogenic bacteria in human medicine. From a practical perspective, UTI mainly caused by uropathogenic E. coli (UPEC) is easy to recur. For such a stubborn UTI infection, a phage designed to destroy UPEC is one potential treatment. We propose to assemble larger genomes required for an entire phage that can be programmed to target specific pathogenic agents and pathological mechanisms, without significant impairment of the phage's ability to replicate. Chemical synthesis of G4 is the first attempt but there are still many challenges. Meanwhile, a lot of work still needs to be done to test the relevant theories and principles. However, the oligonucleotide synthesis combined with the modifications of a phage genome described here will serve as a basis for understanding the procedure. Given the synthetic approaches available and the present knowledge of phage biology, this new strategy may have potential use in antimicrobial therapies. Whole G4 genome assembly will further enhance the role of a synthetic approach for medical application. This new field of research needs to be explored thoroughly in order to meet the present and future medical demands.

## Materials and Methods

### Chemical synthesis of genomes

The oligonucleotides were designed to generate a syn-G4 genome according to the sequence reported by Godson [9]. According to the reference sequence, we mutated T-A, T-G and T-C at the position of 3016 bp 3244 bp and 3328 bp respectively to produce three novel restriction endonuclease sties (*NcoI*, *KpnI* and *EcoRI*, respectively) in order to distinguish from the wild type G4 genome. The full-sized genomes of bacteriophage G4 and m-G4 were synthesized and provided by Sangon Biotech Co., Ltd (Shanghai). Basically, for synthesis of the full-sized genomic DNA, each of syn-G4 and m-G4 genomes was divided into 4 segments, including A,B1,B2,and B3(syn-G4: 5996A: 925 bp, 5996B1:1410 bp, 5996B2: 1250 bp, 5996B3 2091 bp;m-G4: 5996A: 925 bp, 5997B1:1410 bp, 5996B2: 1250 bp, 5996B3 2091 bp). All oligonucleotides were chemically synthesized by Sangon Biotech. The resultant fragments of various lengths with overlap were added together and finally assembled into full-sized genome by direct assembly PCR and ligase chain reaction (LCR) followed by fusion PCR with flanking primers from short overlapping synthetic oligonucleotides. The linear full-sized genomic DNA was circularized by means of enzymatic ligation and the use of preexisted *PstI* restriction sites for preparation of sticky ends using recommended conditions. Transfection of cells with the circular synthetic genome resulted in appearance of infective plaques.

### Circularization of linear genome DNA molecules

PCR products of full-sized genomes were pooled and confirmed by sequencing. The linear DNA products were cleaved with *PstI* and then circularized by ligation with T4 ligase using recommended conditions. The ligation mixture was stayed overnight at 22°C in preparation for infectivity testing.

### Assay of G4 plaques

One microliter of syn-G4 ligation product was electroporated into DH5α cells (Biononatech Co., Ltd, Shenzhen, China), immediately diluted with 600 µl of liquid LB medium, and then divided into two screwcapped glass culture tubes. The two tubes were rotated at 37°C for 60 min and then 200 µl of culture fluid was stained to LB medium A to stay overnight at 37°C. Another microliter of m-G4 and pure water underwent the same procedure and each of 200 µl of culture fluid was stained to LB medium B and C respectively, to stay overnight at 37°C. Phage plaques in A and B were visualized after 6–12 h of incubation at 37°C and recorded by photography at 8 h. No plaque was observed in C.

### Electron microscopy

Several plaques were picked up to 1.5 ml Eppendorf and 0.5 ml of 2.0% (w/v) glutaraldehyde was added for fixation before examination. A concentrated phage sample was negatively stained with 0.2% (w/v) phosphotungstic acid on a carbon-coated grid and examined by JEM100CXII (Japan) transmission electron microscope (Electron Microscope Laboratory, School of Life Sciences, Sun Yat sen University, Guangzhou) at an accelerating voltage of 80 kV. Electron micrographs were taken at a magnification of 800,000×. The phage size was determined from the average of two independent measurements (Anteroposterior diameter and left-right diameter).

### Plaques purification and assay of infectivity

A confluent plaque was picked up and covered with 50 µl SM buffer (50 ml 1 M tris-cl(PH 7.5), 2 g MgSO4, 5.8 g NaCl, 5 ml 2% gelatin and ddH_2_O up to 1 L), gently shaken and maintained in the refrigerator at 4°C for 3 min. After adding a few drops of chloroform, the plate is manually shaken. The phage suspension was centrifuged (5000×g for 10 min) to remove DH5α and debris. The 200 µl of the supernatant was retrieved and diluted to the proper concentration (10^−7^/L∼10^−5^/L) for infectivity test. E. coli strain BL21 (Biononatech Co., Ltd, Shenzhen, China) colony was picked up to 5 ml LB culture medium to prepare log phase of BL21. BL21 were grown in LB broth without antibiotic selection at 37°C until an OD600 of 0.4 was reached. Bacterial cells were diluted in LB broth to a final density of 10^6^ CFU/ml. An aliquot of cells (10^5^ CFU, 100 µl) was added with 100 µl phage suspension with a diluted concentration of 10^−5^/L and mixed with 0.7% soft agar, slightly shaken, then the mixture was plated on LB plates, following overnight incubation at 37°C. Phage plaques were visualized after 6∼18 h of incubation at 37°C.

### PCR assay and DNA sequencing

Specific primers for syn-G4 and m-G4 were synthesized by Jierui Biotech co., Ltd. (Guangzhou), and their sequences were listed in Table 1. The expected length of the amplification product was 443 bp, 603 bp and 645 bp respectively. Plaques purified were picked directly into 50 µl PCRs respectively. Each reaction mixture contained 2.5 of phage suspension, 1.5 µl of 25 mmol/L MgCl_2_, 2 µl of 2.5 mmol/L dNTP, 1.0 µl of each 20 pmol/L primer, 0.4 µl of 5 U/L Taq polymerase (Takara), 25 of primerstar, and ddH_2_O up to total 50 µl. PCR of NLE-pCMV10 for NLE gene was also performed as the control group (date not shown). w The conditions for PCR were as follows: 99°C for 2 min, followed by 30 cycles of 98°C, 10 s, 68°C, 40 s, 72°C, 10 min and a final extension of 72°C for 7 min. The PCR was performed as previously described. The PCR products were analyzed using a Rapid Agarose Gel Electrophoresis System (Wealtec Corp, Sparks, Nev) in 1.0% agarose gels in 5.0 µl loading buffer (10 min at 90 V). The genome was gel-purified and sequenced on a 3730 XL sequencer (3730 XL sequencer, ABI, Sangon) with depth of 2-fold. Sequencing of the cloned amplification product confirmed that it was identical to part of the syn-G4 and m-G4.

**Table 1:** Primers for specific PCR.

Target	Sequences	PCR Product (bp)
Syn-G4	5′-CAAAAATCTTGGAGGAGTCAACTATGAAGTCTC-3′	443
	5′-GGAGTACCGGACTGCGATGGGCATAGAGTAAC-3′	
Syn-G4	5′-GCCTACGGAGATACTCGAGTCTCCGATAC-3′	603
	5′-GGTTGAAGGACGGTTGCTTCACGGTTTAC-3′	
Syn-G4	5′-TCTATATCCCACACCGTCATATCTACGGTC-3′	645
	5′-TAATTACGCGATGCTCAGGAACATAGAAG-3′	
m-G4	5′-CAAAAATCTTGGAGGAGTCAACTATGAAGTCTC-3′	443
	5′-GGAGTACCGGACTGCGATGGGCATAGAGTAAC-3′	
m-G4	5′-GCCTACGGAGATACTCGAGTCTCCGATAC-3′	603
	5′-GGTTGAAGGACGGTTGCTTCACGGTTTAC-3′	
m-G4	5′-TCTATATCCCACACCGTCATATCTACGGTC-3′	645
	5′-TAATTACGCGATGCTCAGGAACATAGAAG-3′	

### Restriction analyses

For restriction analyses, 10 µl of PCR products with expected length of 645 bp were digested with restriction endonucleases (*EcoRI*, *KpnI* and *NcoI*) according to the supplier's recommendations (Takara, Dalian, China). Digestion of syn-G4 and m-G4 with 2.0 µl of *EcoRI* restriction endonuclease (Takara, Dalian) was done at 37°C in 10 m M Tris-HCl, 50 mM NaCl, 15 mM MgCl2, pH 7.5. DNA was digested with 2.0 µl of *KpnI* endonuclease (Takara, Dalian) at 37°C in 10 mM Tris-HCl, 10 mM MgCl2, 0.02% Triton X-100, 0.1 mg/ml BSA. Digestion of DNA with 2.0 µl of *NcoI* restriction endonuclease (Takara, Dalian) was done at 37°C 10 mM Tris-HCl, 50 mM KCl, 1 mM DTT, 0.1 mM EDTA, 0.2 mg/ml BSA and 50% glycerol. The digested DNA was examined by gel electrophoresis after 10 hours digestion. The resultant fragments were separated and purified as described above.

### Gel electrophoresis

DNA was analyzed using a 6.0% (w/v) agarose gel in TAE buffer (40 mmol l^−1^ Tris acetate; 2 mmol L^−1^ EDTA, pH 8.3) with 0.5 µmg ml^−1^ ethidium bromide. Electrophoresis was performed at constant voltage 90 V) for 30 min and visualized by UV light (300 nm) after staining with ethidium bromide (1 Ag/ml). This procedure was followed for all the experiments except where stated differently. The relative sizes of the DNA fragments were estimated by comparing their electrophoretic mobility with that of the standards run with the samples on each gel (D2000marker).

### Theoretical considerations

In order to accomplish the synthesis of infectious G4 genome, we had considered the replication mechanism of G4 [18] and analyzed the methodology of chemical synthesis of genome [19]–[21]. When the sequence of phage G4 was determined by Godson, its structure was almost understood. During the stage of phage G4 replication, there was a circular double-stranded DNA called replication form (RF form) [22]. It was not yet possible for us to synthesize entire genome as long continuous strands of DNA. In designing the oligonclotiedes sets, we adopted the basic methods available for assembling long DNA sequences and the strategy of dividing the genome into 4 segments. Each segment was also divided into 2 or 3 shorter pieces (data not shown). These entire short custom-made single-stranded DNA with overlap were assembled from the oligonucleotides. These segments were finally assembled into full-sized genome by direct assembly PCR and ligase chain reaction (LCR) [21]. These synthetic phage G4 was referred to the G series of X-like phages. They were subsequently grown and handled by all of the methods commonly used for ΦX174 [7]. The linear G4 molecules were then circularized to form a circular RF form after gel-purified using recommended conditions [2].

## Supporting Information

Material S1 **Full-sized genomes of syn-G4 and m-G4 molecules.**
(DOC)

File S1 **Trace files for specific PCR fragments, syn-G4 genome and m-G4 genome.**
(RAR)
