# Supplementary material for: Chemical Synthesis of Bacteriophage G4
Source: PLoS One. 2011 Nov 16;6(11):e27062. doi: 10.1371/journal.pone.0027062 (PMC3217949; doi:10.1371/journal.pone.0027062)
Supplement: Material S1 — Full-sized genomes of syn-G4 and m-G4 molecules. (DOC) [file pone.0027062.s001.doc]

**Supplemental Material**

The oligonculeotides were pooled and gel-purified by Sangon Biotech as described in Materials and methods. The sequences of the short custom-made segments were consistent with the results from both sequencing and agarose gel. Full-sized genomes of syn-G4 and m-G4 molecules were completed and provided by Sangon Biotech. Co., Ltd (Shanghai).


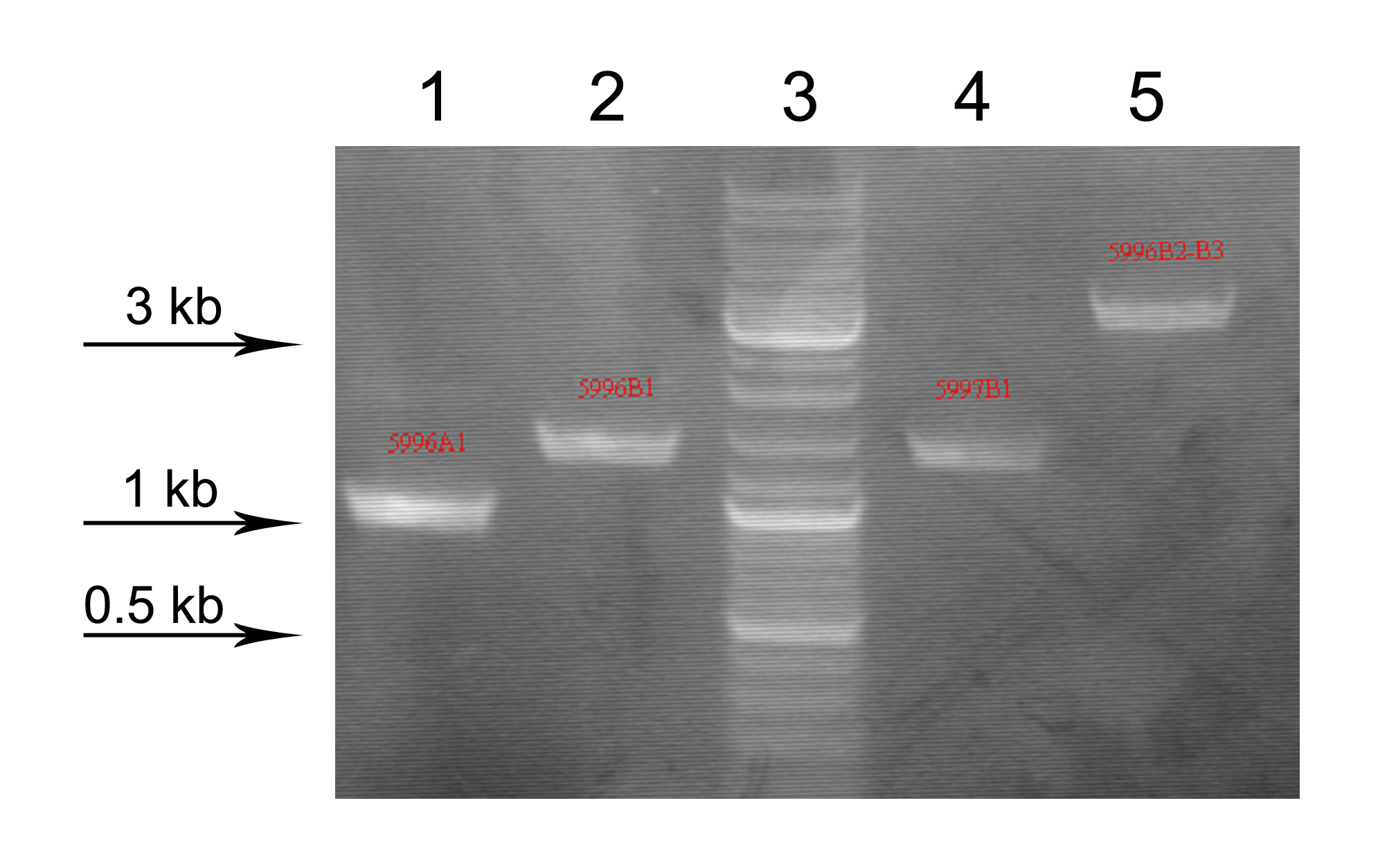


Fig. 1 PCR results of short custom-made segments for syn-G4 and m-G4. Lane 1 and 5, segments shared by both of syn-G4 and m-G4; Lane 2: segment for syn-G4; Lane 3: segment for m-G4. Lane 3, GeneRuler™ DNA Ladder Mix ladders.


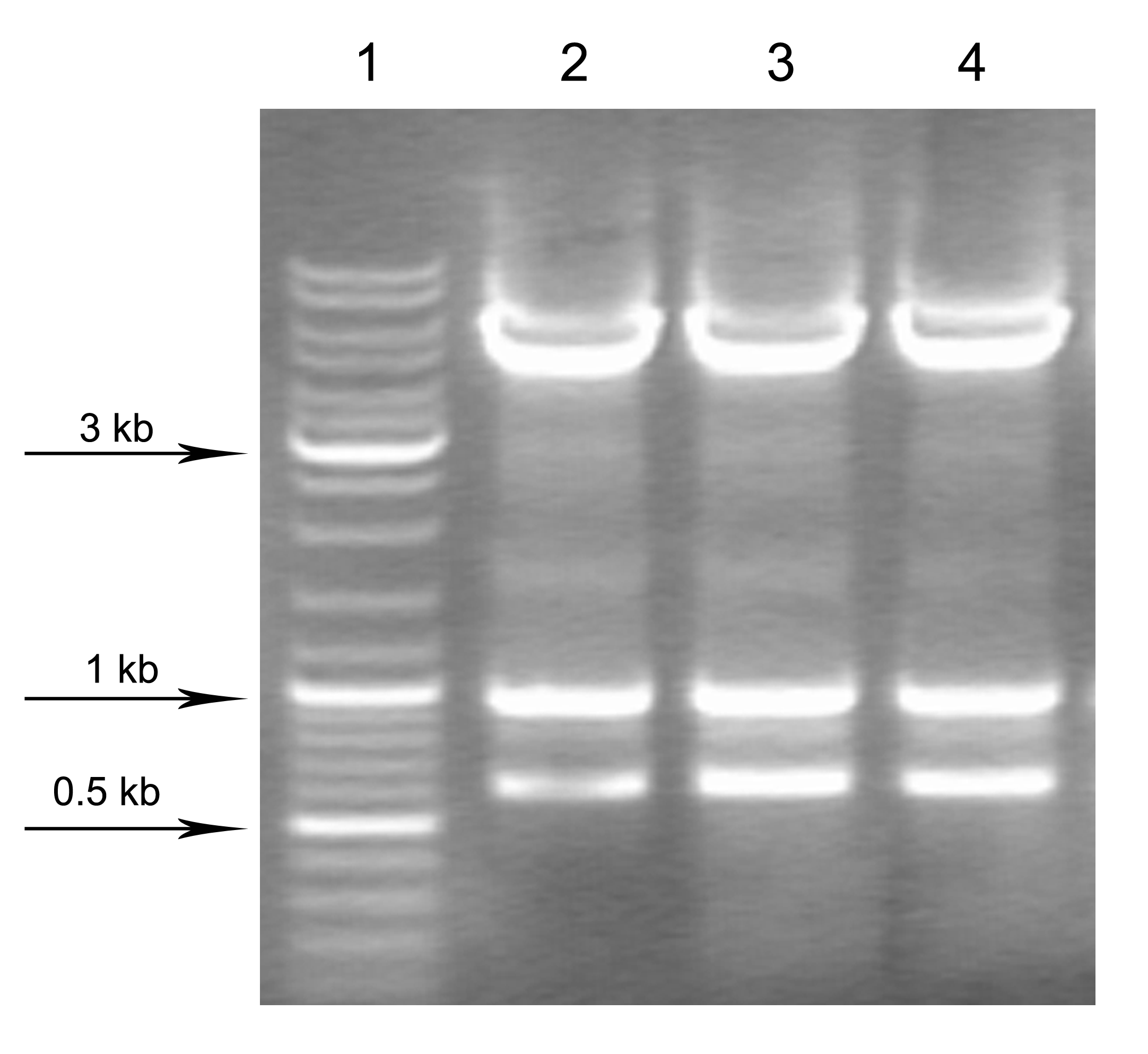


Fig.2 PCR of full-length syn-G4 and m-G4 molecules. Each of the 4 segments was sequenced, and then each of the proper segment was ligated to produce the full-length molecules. Lane 2, syn-G4 molecules; Lane 3 and 4, m-G4 molecules. Lane 1, GeneRuler™ DNA Ladder Mix ladders.
